# Supplementary material for: Variation and Interaction of Distinct Subgenomes Contribute to Growth Diversity in Intergeneric Hybrid Fish
Source: Genomics Proteomics Bioinformatics. 2024 Jul 23;22(6):qzae055. doi: 10.1093/gpbjnl/qzae055 (PMC11810642; doi:10.1093/gpbjnl/qzae055)
Supplement: qzae055_Supplementary_Data [file qzae055_supplementary_data.zip › supplementary material captions-done.docx]

**Supplementary material**

**Figure S1 Appearance of goldfish (2n = 100), common carp (2n = 100), their reciprocal diploid hybrids (2nRC and 2nCR, 2n = 100), their allotetraploid progenies (4nR_2_C_2_, 4n = 200), and the four allotriploid varieties (3nR_2_C, 3nCR_2_, 3nC_2_R, and 3nRC_2_, 3n = 150) derived from interploid crossings with their inbred parents**

These individuals were bred for 24 months after hatching (average size in each hybrid variety).

**Figure S2 CNVs in the four triploid individuals**

The ratio changes of allelic copy number in the four individuals of the triploids are shown. For example, the loss events of whole alleles R or C were observed in the 92 contiguous genes of chr19 and 222 contiguous genes of chr34 in 3nR_2_C-3, the 222 contiguous genes of chr34 in 3nR_2_C-4, and the 92 contiguous genes of chr19 in 3nCR_2_-4. The 1:1 ratio of allelic copy numbers is in chr40 of 3nR_2_C-4 and chr19, chr36, chr25, and chr31 of 3nRC_2_-2.

**Figure S3 Distribution of seven allelic expression patterns in reciprocal diploid hybrids (2nRC and 2nCR)**

Red represents the expression of allele R, while blue represents the expression of allele C.

**Figure S4 Gene modules correlated with growth in the hybrid varieties**

**A.** Correlation between coexpressed modules and sampling traits. Heatmap color represents correlation coefficients (“*” represents Fisher’s asymptotic *P* < 0.05; “**”, *P* < 0.01; “***”, *P* < 0.001). Rows are different modules, and columns are different growth traits. **B.** Distribution of gene significance and module membership of ME01. ME01, Module Eigengene 1.

**Figure S5 Correlational analyses between the expression of genes in subgenome C (21 growth-regulated genes) and body weight**

**A.** Heatmap exhibiting the expression of genes in subgenome C across the 29 individuals (eight months after hatching). The distribution of PCC values in the 21 genes is shown. **B.** The two groups of the five hybrids were classified based on body weight (8 months after hatching) and gene expression.

**Figure S6 Distribution of SSGs and AGPs of the 3693 growth-regulated genes**

SSGs, species-specific genes.

**Figure S7 Growth-regulated genes with strong positive correlation (PCC > 0.5) between expression and body weight**

There are 833 genes in 2nRC, 812 genes in 2nCR, 1315 genes in 3nR_2_C, 1182 genes in 3nCR_2_, 666 genes in 3nRC_2_, and 1149 genes in 3nC_2_R.

**Table S1 Sampling information for the hybrid varieties originating from hybridization of goldfish (2nRR) and common carp (2nCC)**

**Table S2 Summary of WGS and transcriptome data for 29 individuals**

**Table S3 Genotypes of hybrid fish inferred from WGS data**

**Table S4 Summary of mitochondrial reads mapped to goldfish and common carp genomes in transcriptome data (29 individuals)**

**Table S5 Transcriptome-based mitochondrial type of hybrid fish (29 individuals)**

**Table S6 Overview of CNVs predicted from WGS data**

**Table S7 Summary of allelic copy numbers inferred from WGS data**

**Table S8 Overview of transcriptome data from 131 individuals in the six hybrid varieties**

**Table S9 Summary of gene expression changes regulated by mitochondrial genetics**

**Table S10 Reversed expression correlations in the two reciprocal F_1_ hybrids**

**Table S11 Overview of differential DNA methylation between 3nR_2_C and 3nRC_2_ in 3693 growth-regulated genes**
