# Supplementary figures and images for: Variation and Interaction of Distinct Subgenomes Contribute to Growth Diversity in Intergeneric Hybrid Fish
Source: Genomics Proteomics Bioinformatics. 2024 Jul 23;22(6):qzae055. doi: 10.1093/gpbjnl/qzae055 (PMC11810642; doi:10.1093/gpbjnl/qzae055)

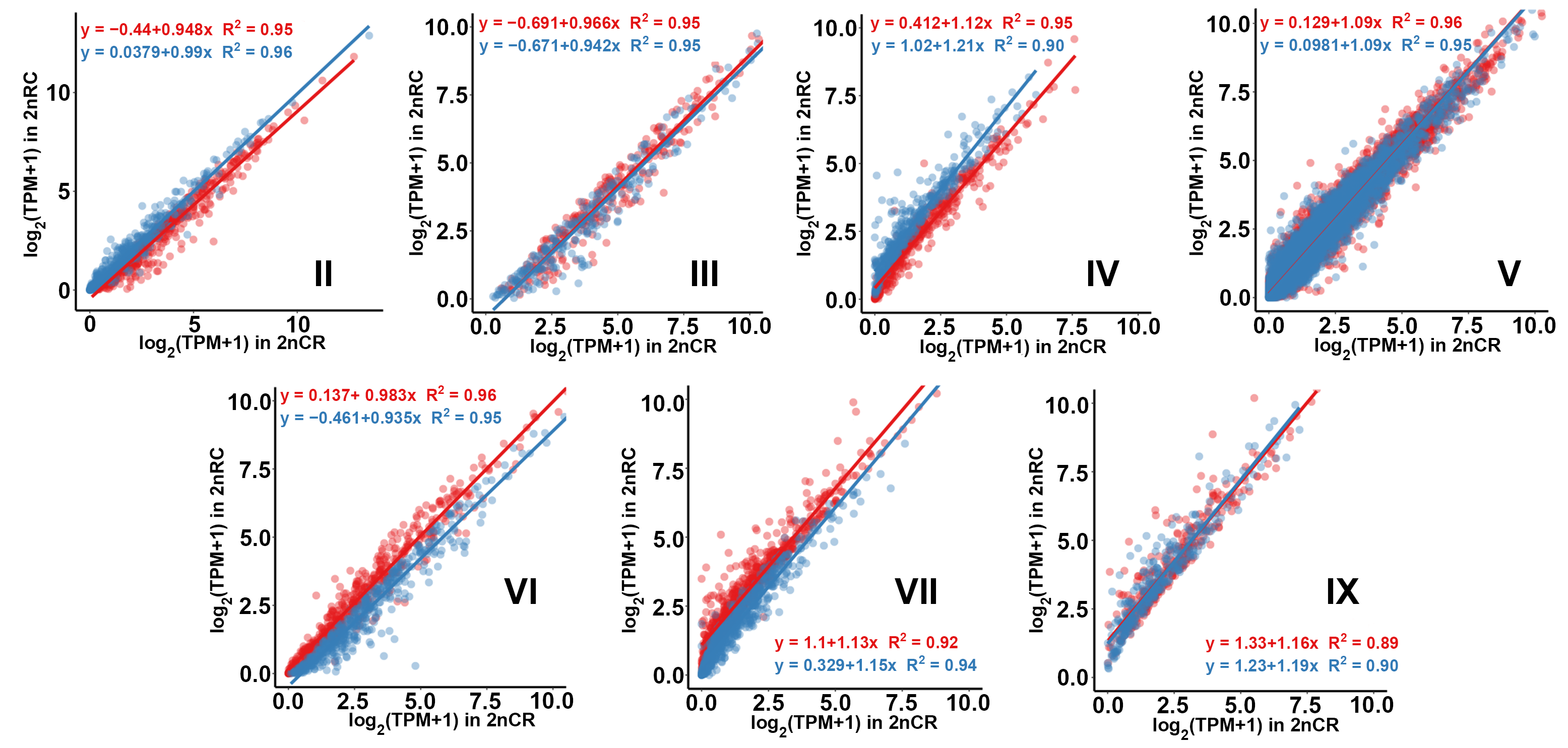

Supplement: qzae055_Supplementary_Data [file qzae055_supplementary_data.zip › Figure S3.tif]

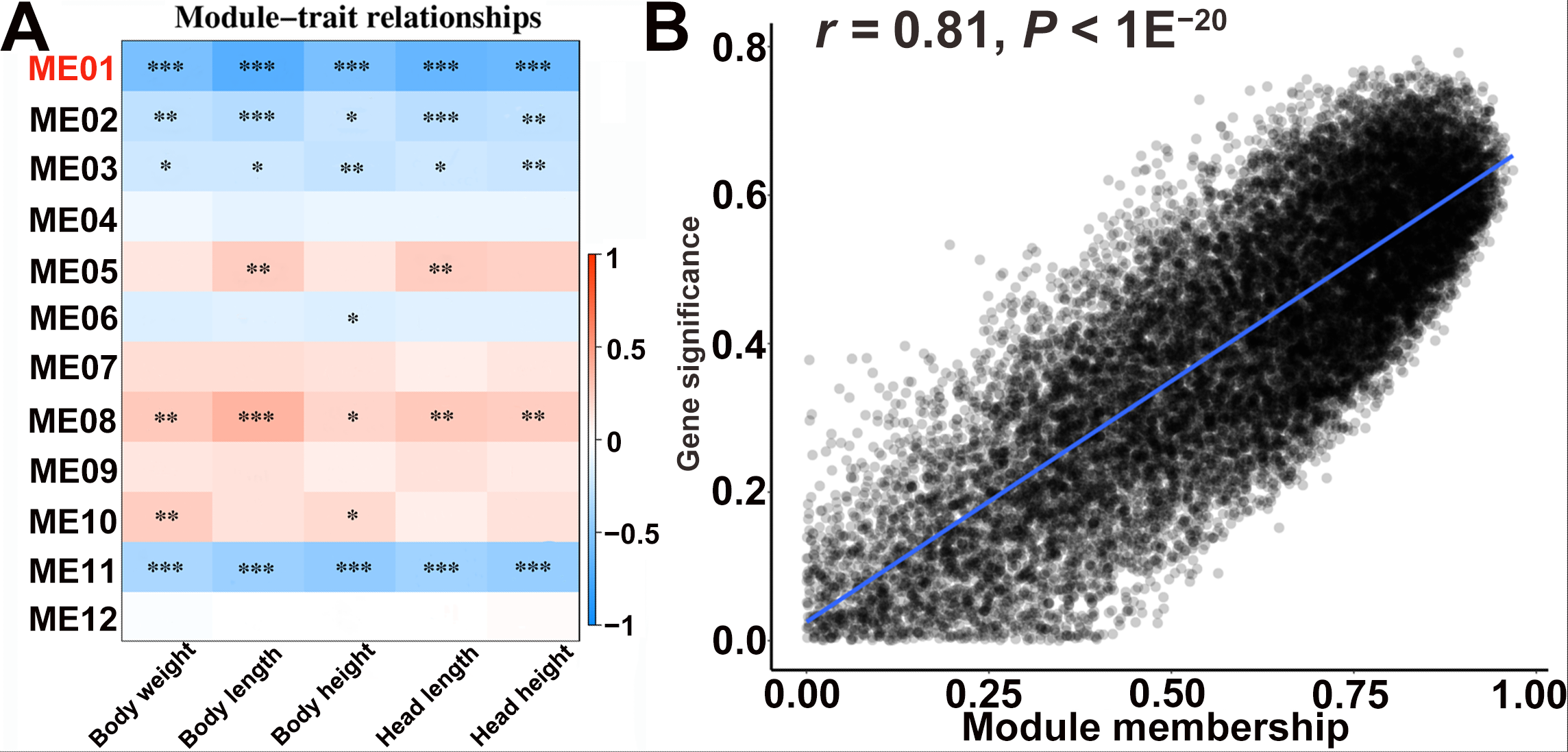

Supplement: qzae055_Supplementary_Data [file qzae055_supplementary_data.zip › Figure S4.tif]

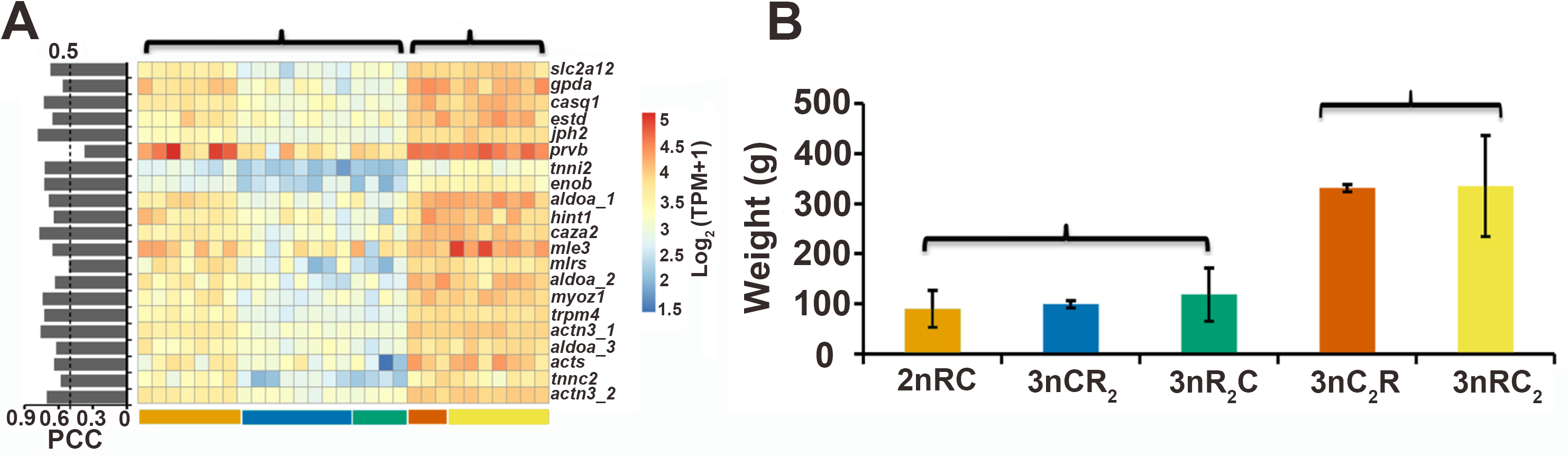

Supplement: qzae055_Supplementary_Data [file qzae055_supplementary_data.zip › Figure S5.tif]

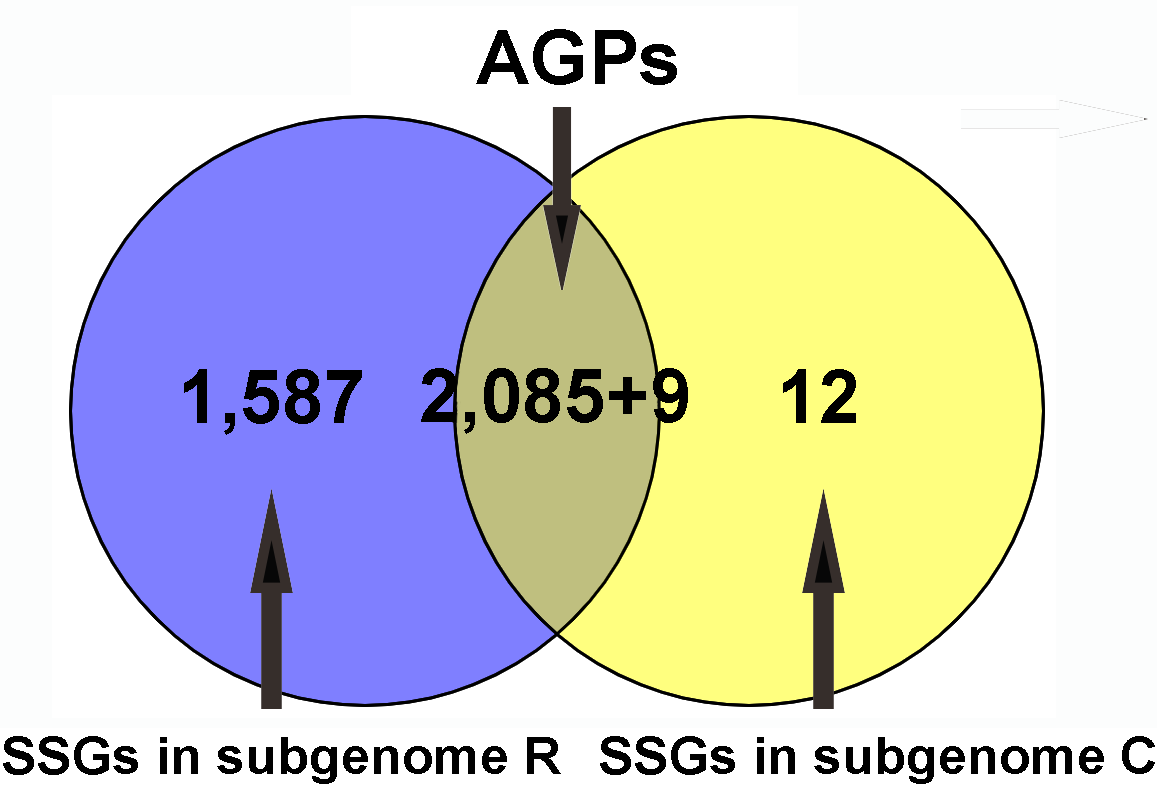

Supplement: qzae055_Supplementary_Data [file qzae055_supplementary_data.zip › Figure S6.tif]

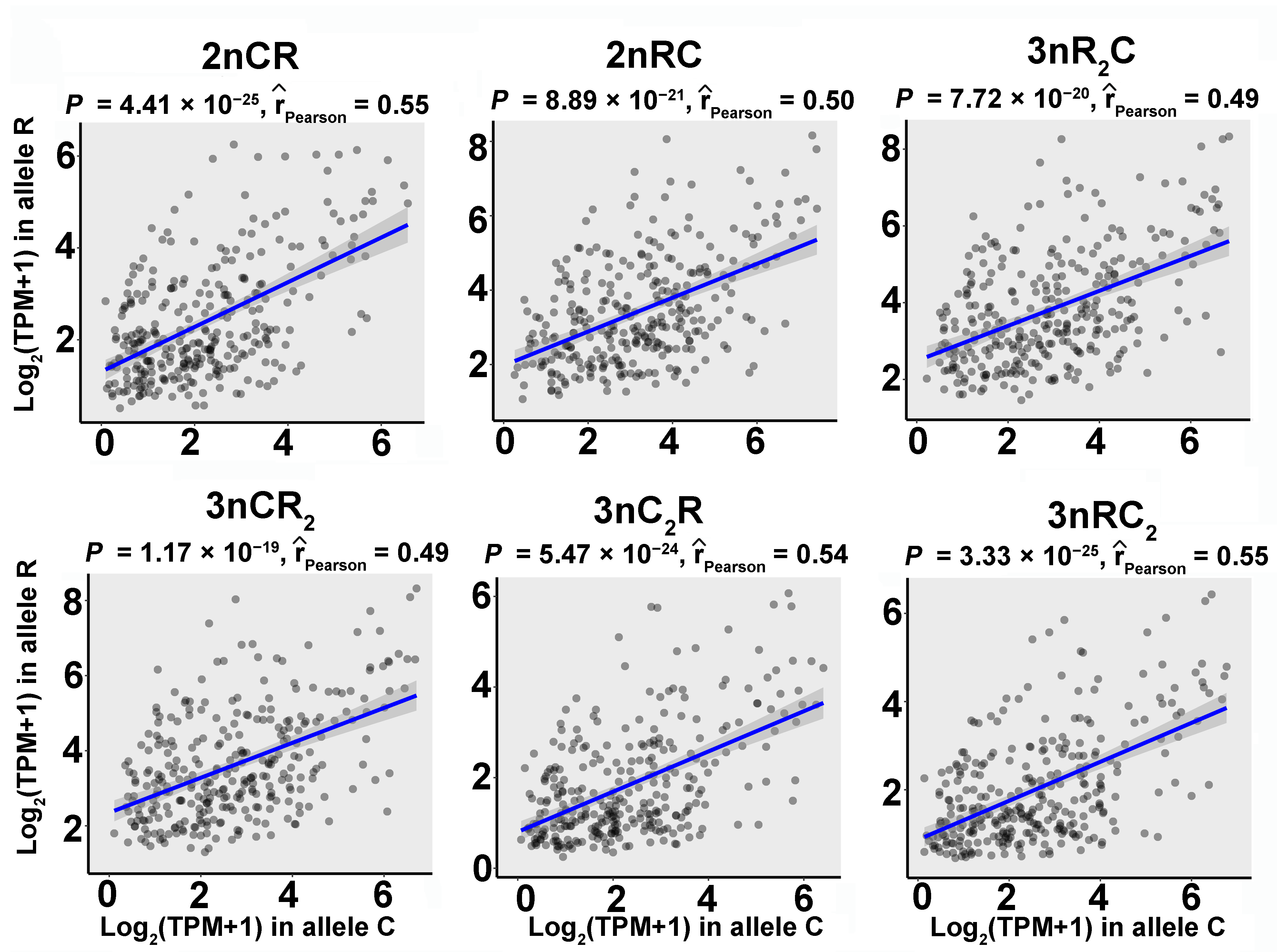

Supplement: qzae055_Supplementary_Data [file qzae055_supplementary_data.zip › Figure S7.tif]

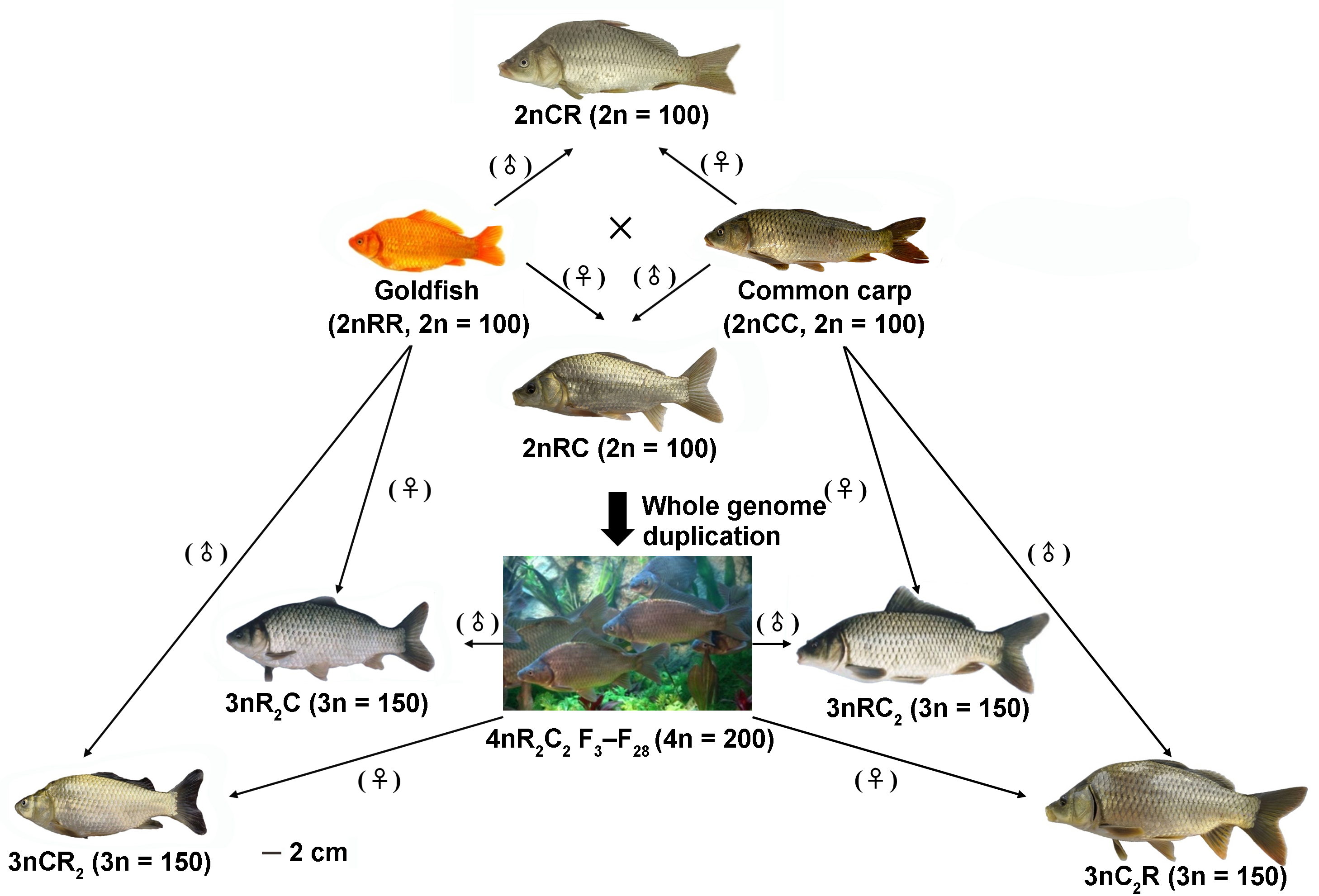

Supplement: qzae055_Supplementary_Data [file qzae055_supplementary_data.zip › Figure S1.tif]

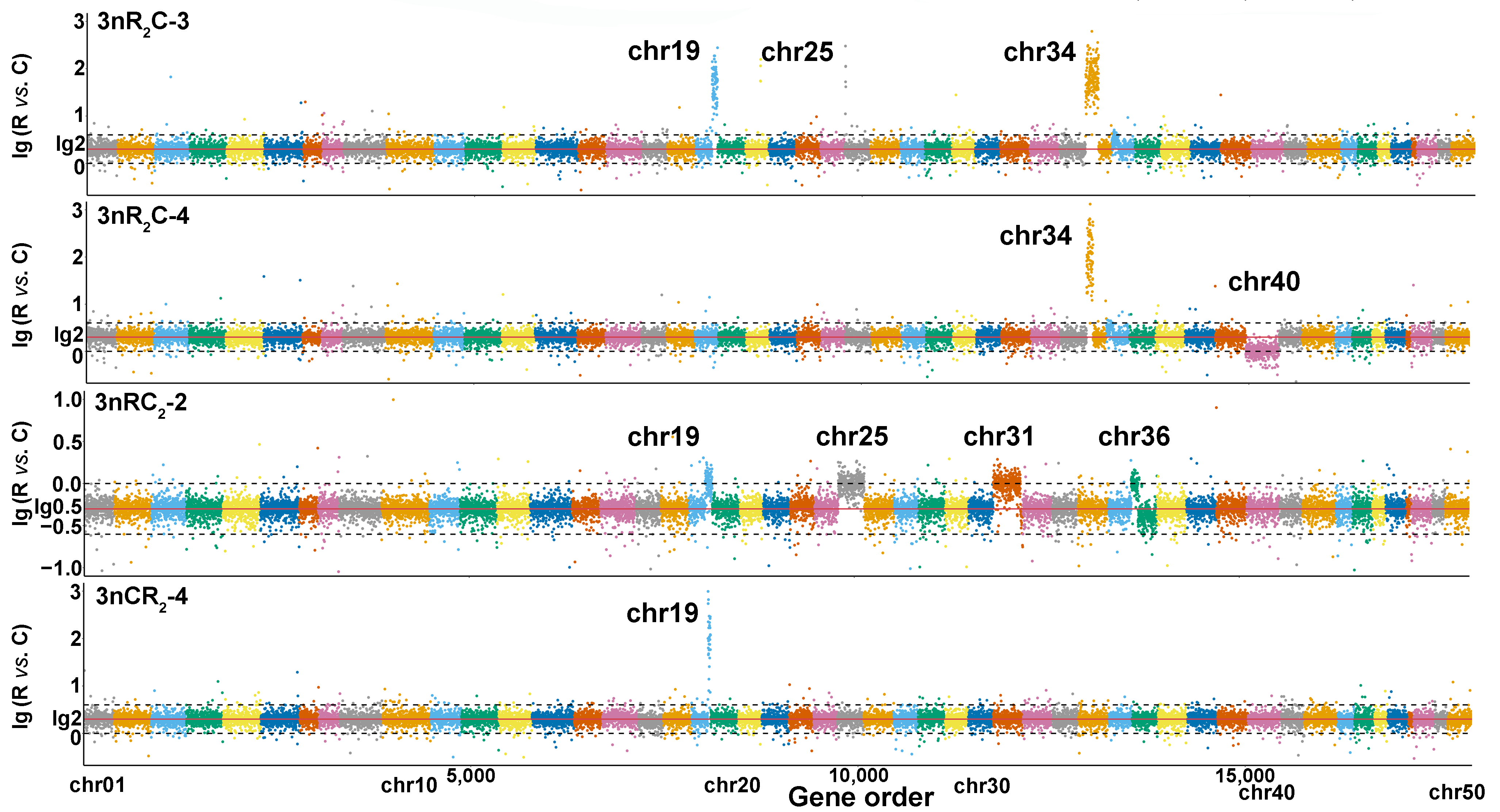

Supplement: qzae055_Supplementary_Data [file qzae055_supplementary_data.zip › Figure S2.tif]
